# Enhancing hydrogen generation from sodium borohydride hydrolysis and the role of a Co/CuFe_2_O_4_ nanocatalyst in a continuous flow system

**DOI:** 10.1038/s41598-024-60428-5

**Published:** 2024-04-26

**Authors:** Faezeh Mirshafiee, Mehran Rezaei

**Affiliations:** School of Chemical, Petroleum and Gas Engineering, Iran University of Science and Technology (IUST), Tehran, Iran

**Keywords:** Hydrogen, Hydrolysis, NaBH_4_, Ferrite, Sol–gel, Continuous, Hydrology, Materials science

## Abstract

In this study, a series of cobalt-based spinel ferrites catalysts, including nickel, cobalt, zinc, and copper ferrites, were synthesized using the sol–gel auto-combustion method followed by a chemical reduction process. These catalysts were employed for accelerating hydrogen generation via the sodium borohydride hydrolysis process. A continuous stirred tank reactor was used to perform catalytic reactor tests. All samples were subjected to analysis using XRD, FESEM, EDX, FTIR, and nitrogen adsorption–desorption techniques. The results revealed that the cobalt-based copper ferrite sample, Co/Cu-Ferrite, exhibited superior particle distribution, and porosity characteristics, as it achieved a high hydrogen generation rate of 2937 mL/min.g_cat_. In addition, the higher electrical donating property of Cu-Ferrite which leads to the increase in the electron density of the cobalt active sites can account for its superior performance towards hydrolysis of NaBH_4_. Using the Arrhenius equation and the zero-order reaction calculation, activation energy for the sodium borohydride hydrolysis reaction on the Co/Cu-Ferrite catalyst was determined to be 18.12 kJ/mol. This low activation energy compared to other cobalt-based spinel ferrite catalysts confirms the catalyst's superior performance as well. Additionally, the outcomes from the recycling experiments revealed a gradual decline in the catalyst's performance after each cycle during 4 repetitive cycles. The aforementioned properties render the Co/Cu-Ferrite catalyst an efficient catalyst for hydrogen generation through NaBH_4_ hydrolysis.

## Introduction

Recent population growth and industrialization have increased energy demand. Currently, the majority of energy is sourced from non-renewable fossil fuels which are harmful to the environment and cannot be replenished. Hence, nations worldwide are prioritizing the reduction of fossil fuel consumption while increasing the production of renewable energy sources^1^. Recently, hydrogen has attracted significant attention worldwide as a clean energy carrier in developing renewable energy sources. Hydrogen is an attractive alternative fuel source due to its lack of pollution, as its only byproduct is pure water. Additionally, hydrogen possesses a notable gravimetric energy density of approximately 120 MJ/kg, a value that surpasses that of many conventional fuels. Moreover, the versatility of hydrogen makes it a valuable tool in addressing energy and environmental challenges, as hydrogen can be produced from various sources, including renewable sources such as wind and solar power^2^. In addition, hydrogen can be produced through biological means by utilizing industry-based or agricultural wastes^3^. To promote a hydrogen-based society, it is necessary to seek clean and safe methods for hydrogen production. One of the novel technologies for hydrogen production is the use of hydrogen storage materials. Metal hydrides are considered to be secure materials for hydrogen storage in the solid state, as they offer high volumetric densities and can operate under mild conditions^4^. They can produce hydrogen and release it to the system when needed without the need for storage. Sodium borohydride has attracted significant attention among various hydrides due to its non-explosive nature, high hydrogen storage capacity, etc.^5^. Borohydride hydrolysis can serve as a viable source of hydrogen that can be readily supplied to Proton Exchange Membrane Fuel Cells (PEMFC) to power electronic devices, including but not limited to vehicles, smartphones and tablets^6^. However, the slow kinetics of the sodium borohydride hydrolysis process poses a challenge. Therefore, the design of an efficient catalyst is inevitable. Numerous metal nanoparticle catalysts like Co^7^, Ni^8^, Fe^9^, mixed metals^10^, etc. have been developed for this process. Nonetheless, the high surface energy of these nanoparticles results in their agglomeration^11^. Thus, the utilization of catalytic support materials was proposed as a solution to mitigate this issue. To date, various support materials, such as metal oxides^12^, carbon-based materials^13^, zeolites^14^, MOF^15^, etc. have been suggested.

Spinel ferrites, with the general formula MFe_2_O_4_, are a class of magnetic spinel materials. Due to their high electrical resistance, permeability, magnetization, and thermodynamic stability, they are suitable for a wide range of applications, including electrical devices, medical science, microwave absorption, water purification, etc.^16^. Recently, the spinel ferrites were also used as a catalyst in a few sodium borohydride hydrolysis process studies. Wang et al.^17^ evaluated CoFe_2_O_4_ as a catalytic support in the hydrolysis of sodium borohydride and modified it with transition metals such as ruthenium, palladium, rhodium, platinum, iridium, and silver. The results showed that ruthenium supported on cobalt ferrite with TOF (Turnover frequency) of 421 mol_H2_/min.mol_cat_ showed the best catalytic performance. Liang et al.^18^ developed a NiB/NiFe_2_O_4_ catalyst for hydrogen generation from NaBH_4_ hydrolysis. Using this catalyst, a hydrogen generation rate (HGR) of 299.88 mL/min.g_cat_, higher than pure NiB, has been achieved. Abdelsalam et al.^19^ investigated Ag/CoFe_2_O_4_–CNT performances in the hydrolysis of sodium borohydride at room temperature. The results revealed the H_2_ generation rate as high as 320 mL/min.g_cat_, and a low activation energy of 14.7 kJ/mol. Zhang et al.^20^ synthesized CuFe_2_O_4_ through a one-pot approach, which was utilized as a catalyst in the production of hydrogen via the hydrolysis of sodium borohydride. The CuFe_2_O_4_ nanocubes achieved a high hydrogen generation rate of 1.5 L/min.g_cat_ which was higher than other shaped CuFe_2_O_4_ materials.

Given the promising potential of these nanomaterials, exploring various spinels as a support is an intriguing avenue for further investigation. To date, several methods have been utilized for the preparation of spinel ferrites, including the sol–gel auto-combustion method^21^, solvothermal techniques^22^, microwave-assisted methods^23^, and mechanical approaches^24^. Inbaraj et al. conducted a study where they synthesized CoFe_2_O_4_ nanomaterial using a green synthesis method that involved natural honey. In this method, honey acted as a fuel or reducing agent for the sol–gel auto-combustion process. The hydroxyl and amine groups present in honey provided the initial molecular matrix for Co^2+^ and Fe^3+^ ions^25^. A study conducted by G et al. involved the synthesis of nanoparticles of Ni_x_Mg_1-x_Fe_2_O_4_(x = 0, 0.2, 0.4, 0.6). The nanoparticles were created through combustion with the assistance of microwaves. The process used Tamarindus indica seed extract as fuel. The resulting nanoparticles were cubic and had an average crystalline size of 17–18 nm^26^. Nguyen et al. studied the synthesis of ZnFe_2_O_4_@ZnO nanocomposites using Chrysanthemum spp. floral waste as a green method. Floral waste was used as the reducing and stabilizing agents during the conversion of metal ions into metal oxide and so during the biosynthesis of ZnFe_2_O_4_@ZnO nanocatalyst^27^. Each method mentioned for preparing spinel ferrites has yielded distinct and noteworthy results. Among these methods, the sol–gel auto-combustion approach is advantageous due to its ease, safety, and rapid production, resulting in time, and cost savings. Moreover, this method offers several advantages, over the other methods. It allows for easy control of the particle size of the product, ensures chemical homogeneity and purity in the final product, and facilitates the formation of extended networks by partially hydrolyzed species, which lowers the crystallization temperature^28^. Additionally, compared to other methods, the auto-combustion method is a time and energy-efficient approach that can be easily scaled up for complex oxide preparations^29^. Sol–gel auto-combustion method involves using metal nitrates and organic fuels like citric acid, glycine, urea, etc. as precursors. The fuel acts as a reducing agent and also provides the energy for the combustion reaction. The process involves mixing precursors in a solution and heating them. Due to the presence of fuel and oxidizer, coupled with highly exothermic reactions, the mixture can lead to an auto-combustion reaction, causing the rapid conversion of gels into ash and gaseous by-products. The ash left behind after the combustion process mainly consists of metal oxides, which are then calcined to obtain the desired catalysts^30^. Accordingly, in the present study, we have employed the sol–gel auto-combustion method to synthesize cobalt, copper, nickel, and zinc-based spinel ferrites as catalytic support. Following this, we have deposited the active phase of cobalt onto these supports to utilize them in the hydrolysis process of sodium borohydride. To scale up the process, it is necessary to conduct hydrogen generation in a continuous flow system. In addition, continuous flow systems offer several advantages over batch reactors. Continuously Stirred Tank Reactors (CSTR) operate under steady-state conditions, offering reproducible results compared to batch reactors where conditions may fluctuate during the reaction cycle. In addition, continuous flow systems provide enhanced control over reaction parameters such as temperature, pressure, flow rates, and residence time. This level of control allows for optimizing reaction conditions to enhance efficiency while improving process safety too. Moreover, the integration of continuous flow systems with analytical techniques enables real-time monitoring of reaction progress, so, facilitating immediate adjustments to optimize the process. Accordingly, a syringe pump was utilized in this study to facilitate continuous hydrogen generation. Ultimately, the effective parameters influencing hydrogen generation rate and kinetic parameters in the continuously stirred tank reactor were identified.

## Materials and methods

### Chemicals

The chemicals used in the study were Fe(NO_3_)_3_.9H_2_O (Merck), Co(NO_3_)_2_.6H_2_O (Merck), Ni(NO_3_)_2_.6H_2_O (Merck), Cu(NO_3_)_2_.3H_2_O(Merck), Zn(NO_3_)_2_.6H_2_O(Merck), Citric acid (Sigma-Aldrich), Amonia(Nanotech), NaBH_4_ (Sigma-Aldrich, 98%), and NaOH(Merck). All chemical materials were of analytical grade and used as received. In addition, for the preparation of all the solutions, deionized water was used.

### Synthesis of catalysts

To synthesize MFe_2_O_4_ (M = Co, Ni, Cu, Zn) nanoparticles, a stoichiometric molar ratio of metal nitrates, M/Fe = 0.5, was initially dissolved in 100 mL of deionized water and stirred for approximately 10 min. Subsequently, citric acid was added to the solution at a 1:1 molar ratio concerning the metals (Fe^3+^ plus M^2+^). The pH of the solution was then adjusted to 7 by adding ammonia solution dropwise. The resulting solution was subjected to stirring at 100°C until it formed a viscous gel. In a short time, the gel underwent auto-combustion and transformed into ash. The combination of highly exothermic gel formation reactions and the presence of fuel contributed to the auto-combustion and rapid transformation of gels into ash in sol–gel systems. The obtained ash was ground and subjected to calcination in air at 600℃ for a duration of 3 h. The chemical reactions involving metal and iron precursors with citric acid can be described as follows:

$${\text{M}}\left( {{\text{NO}}_{{3}} } \right)_{{2}} + {\text{Fe}}\left( {{\text{NO}}_{{3}} } \right)_{{2}} + {\text{nC}}_{{6}} {\text{H}}_{{8}} {\text{O}}_{{7}} \to {\text{MFe}}_{{}} \left( {{\text{C}}_{{6}} {\text{H}}_{{5}} {\text{O}}_{{7}} } \right)_{{\text{n}}} + {\text{2NO}}_{{3}}^{ - } + {\text{H}}_{{2}} {\text{O}}$$

Metal citrate complexes (MFe(C_6_H_5_O_7_)_n_) form a gel-like precursor through hydrolysis and condensation reactions in the presence of citric acid and other reagents. Upon heating, the gel precursor undergoes an auto-combustion process, leading to the decomposition of the gel precursor to the MFe_2_O_4_ phase and the formation of gaseous by-products. For the synthesis of supported catalysts with a 30 wt.% cobalt content, ferrites and Co(NO_3_)_2_.6H_2_O were dissolved in 50 mL of deionized water and subsequently stirred for 10 min. A solution of sodium borohydride was then gradually added dropwise to the aforementioned aqueous solution at a molar ratio of NaBH_4_/Co (II) = 5. Following a mixing duration of 20 min, the precipitate was filtered, washed with deionized water, and dried at 80 °C overnight. To ensure the reproducibility of the outcome, all synthesis procedures were repeated three times.

### Catalytic activity tests

**Figure 1:**
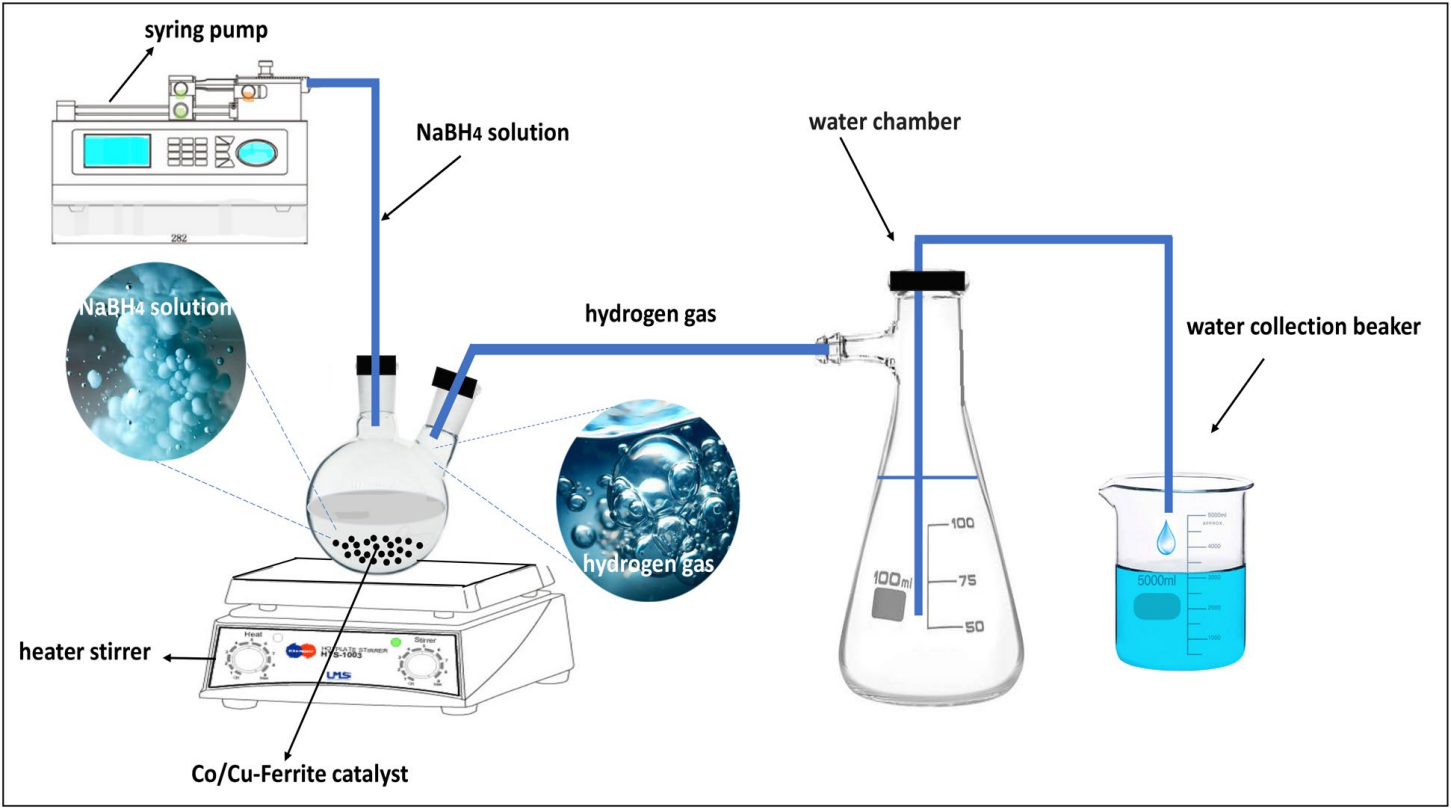
A simple representation of the ongoing continuous experimental setup being utilized.

To determine the rate of hydrogen generation, a hydrolysis reaction was conducted in a continuous system. Initially, 16 mg of catalyst was introduced into the two-neck flask, as a reactor, at a fixed temperature of 35℃. Subsequently, 3 mL of an aqueous solution containing 2wt.% NaBH_4_ and 4wt.% NaOH was injected into the glass reactor using a Terufusion Syringe Pump, model STC 523, with a flow rate of 30 mL/h. The batch hydrolysis process was conducted under the same order and conditions, i.e. temperature of 35 °C, using 16 mg of catalyst and a 3 mL solution containing 2 wt.% NaBH_4_ and 4 wt.% NaOH. The sole difference between these two methods was using a syringe pump with a predetermined flow rate in the continuous stirred tank reactor instead of administering the reagent solution all at once with a regular syringe. The experimental setup for generating H_2_ from the hydrolysis of NaBH_4_ in a continuous system is shown in Fig. 1. H_2_ evolution began immediately after feed introduction and the volume of produced hydrogen was determined by monitoring the displacement of water level in the adjacent vacuum Erlenmeyer.

The hydrogen generation rate (HGR) is calculated using the generated hydrogen volume per reaction time and catalyst amount (having a mL/(minute*g_cat_) unit). However, in this formula, the generated hydrogen volume is the maximum volume produced during a certain time, as reported in the literature^31^. Each experiment was conducted at least three times, and the average values were reported. Following the reaction, the catalyst was separated using a magnet and thoroughly washed with water and ethanol before being dried at 100℃. These catalysts were subsequently subjected to reuse under identical experimental conditions as those utilized previously.

### Characterization

The X-ray diffraction (XRD) spectrum of the synthesized catalysts was determined using a Bourevestnik (model DRON-8, Russian) apparatus. Morphological analysis was conducted via scanning electron microscopy (FESEM) using a TESCAN-MIRA3 (Czech) instrument. The distribution of elements was investigated by energy-dispersive X-ray (EDX) mapping analysis. The surface area and pore size distribution of the prepared samples were ascertained using physical adsorption and desorption of nitrogen on the surface of the catalyst, within the relative pressure range of 0.05 to 0.99. This analysis was conducted using a Belsorp mini (Japan) apparatus.

## Results and discussion

### Samples characterization

#### XRD analysis

**Figure 2:**
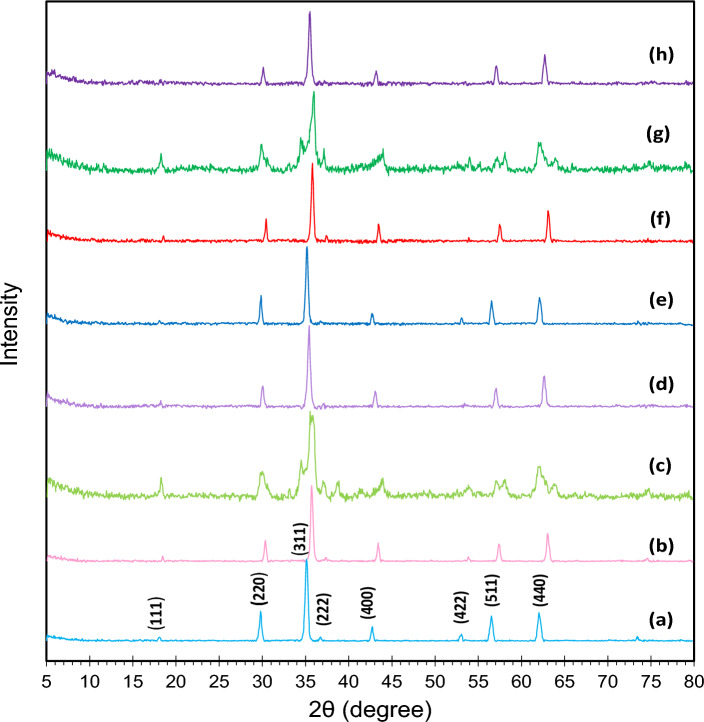
XRD patterns of (**a**) Zn-Ferrite, (**b**) Ni-Ferrite, (**c**) Cu-Ferrite, (**d**) Co-Ferrite, (**e**) Co/Zn-Ferrite, (**f**) Co/ Ni-Ferrite, (**g**) Co/Cu-Ferrite, and (**h**) Co/Co-Ferrite.

Figure 2 displays the XRD spectrum of the synthesized samples. Specifically, the characteristic peaks located at 2θ = 18.5, 29.8, 35.12, 42.72, 53.15, 56.55, and 62.06° in Fig. 2a are attributed to zinc ferrite while the peaks observed at 2θ = 30.29, 35.68, 43.35, 53.88, 57.35, and 63.01° in Fig. 2b are associated with nickel ferrite. In addition, the relatively broad peaks located at 18.27, 29.8, 33.13, 35.52, 37, 38.71, 43.88, 53.85, 58.09, and 62.06° in Fig. 2c and the characteristic peaks at 30.09, 35.46, 43.15, 57.06, and 62.6° in Fig. 2d indicate the formation of copper and cobalt ferrites, respectively. The diffraction peaks can be indexed to the (111), (220), (222), (311), (400), (422), (511), and (440) crystal planes of nanoparticles^32^. Upon the incorporation of cobalt on each of the ferrites as support, Fig. 2e-h, the XRD spectrum was the same, and no discernible peaks associated with cobalt metal were detected, possibly due to its large dispersion. The average crystallite size of Co/Zn-Ferrite, Co/Ni-Ferrite, Co/Cu-Ferrite, and Co/Co-Ferrite, determined by the Scherrer equation, was estimated at 28.1, 36.2, 23.5, and 23.2 nm respectively. Therefore, the examination of the obtained XRD spectra not only confirms the successful synthesis of both the supports and supported catalysts but also demonstrates the absence of impurities in the synthesized catalysts.

#### FESEM analysis

**Figure 3:**
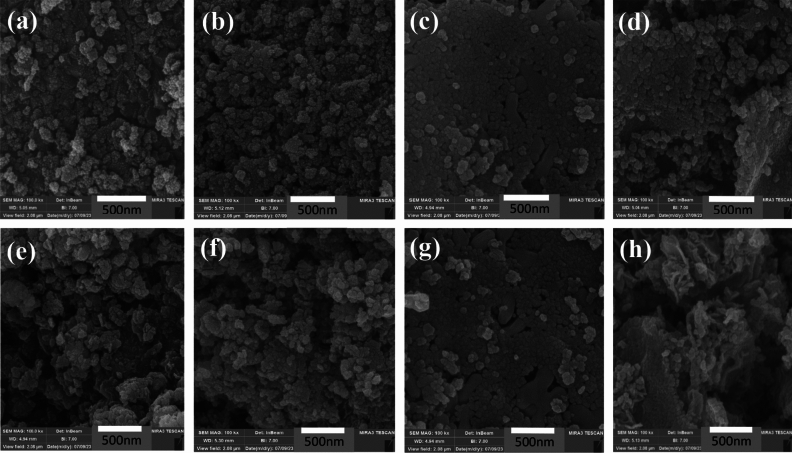
FE-SEM images of (**a**): Ni-Ferrite, (**b**): Co-Ferrite, (**c**): Cu-Ferrite, (**d**): Zn-Ferrite, (**e**): Co/Ni-Ferrite, (**f**): Co/ Co-Ferrite, (**g**): Co/Cu-Ferrite, and (**h**): Co/Zn-Ferrite.

The FESEM technique was employed to ascertain the morphology and dimensions of the particles, as well as to evaluate the distribution of constituents within the synthesized catalysts. The outcomes of this analysis are depicted in Fig. 3. Comparing the micrographs, it is obvious that all the ferrites exhibit polyhedral shapes close to spherical morphology, with an average particle size ranging from 20–100 nm. Among different ferrites, copper ferrite has a smaller particle size, as corroborated by the outcomes reported in the X-ray diffraction analysis. These components are arranged close to each other, forming an intricate network. As evidenced by Fig. 3e–h, following the impregnation of cobalt onto various ferrite supports, the resultant product retains a morphology similar to that of the support, albeit with a marginally rougher surface. This phenomenon can be attributed to the presence of cobalt nanoparticles, which may sometimes aggregate. Upon comparison of the figures, it is evident that among the different supports and supported samples, the most homogeneously dispersed and least agglomerated particles are observed in Cu-Ferrite and Co/Cu-Ferrite samples.

**Figure 4:**
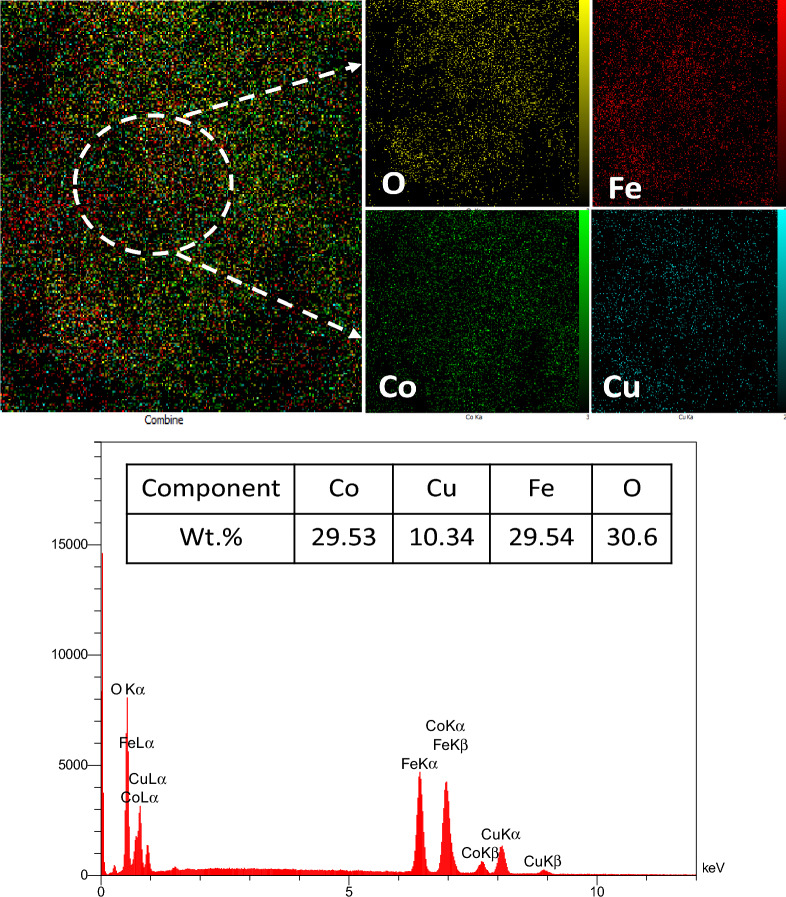
EDS mapping (top), and EDX (down) analysis of Co/Cu-Ferrite.

The EDS mapping analysis was used to validate the presence of four distinct constituents, namely cobalt, copper, iron, and oxygen, in the Co/Cu-Ferrite sample. The result of Fig. 4 revealed that these components are present and uniformly distributed throughout the synthesized sample, with no discernible influence of nanoparticle aggregation. Furthermore, the intensity of the peaks corresponding to the aforementioned elements in the EDX image demonstrated that the cobalt content in the synthesized Co/Cu-Ferrite sample was near the weight percentage employed during the synthesis, thereby affirming the precision of the synthetic methodology.

#### N_2_ adsorption/desorption analysis

To determine the pore characteristics of the catalysts, the BET method was used. This method uses the surface adsorption of nitrogen gas and studies its adsorption and desorption isotherms to provide information about surface area, pore volume, and more.

Table 1 shows that Co-Ferrite and Cu-Ferrite have the highest and lowest surface area and pore volume among different supports. However, after cobalt was loaded onto each of the carriers, an increase in surface area and total pore volume was observed, with Co/Cu-Ferrite having the highest surface area. It is possible that the introduction of cobalt could increase the surface area due to chemical or physical changes in the support. These changes may include oxidation, reduction, the creation of additional surface roughness, defects, active sites, gasification, etc. This could lead to the creation of new pores or the enlargement of existing ones. In addition, when Co nanoparticles are loaded onto the carrier, they can interact with the surface of the support through various mechanisms such as physical adsorption, chemical bonding, or diffusion into the support matrix. These interactions can lead to the formation of a new phase or structure at the interface between Co nanoparticles and the support so, increasing the pore characteristic. A greater surface area means there are more active sites available for reactant molecules. Therefore, based on the results of the FE-SEM and BET method, the Co/Cu-Ferrite sample is expected to perform better in the sodium borohydride hydrolysis process.

**Table 1:** Textural properties of spinel ferrites and supported ferrites.

Sample	S_BET_^a^	V_total_^b^	M.P.D^c^
Ni-Ferrite	15.35	0.10	26.12
Co-Ferrite	26.72	0.18	27.09
Cu-Ferrite	11.35	0.09	31.97
Zn-Ferrite	16.25	0.14	36.88
Co/Ni-Ferrite	25.79	0.19	30.61
Co/Co-Ferrite	30.77	0.25	32.68
Co/Cu-Ferrite	32.65	0.32	39.28
Co/Zn-Ferrite	30.74	0.16	42.70

^a^Surface area with BET Method (m^2^/gr).

^b^Volume adsorbed at p/p_0_ = 0.99 (mL/gr).

^c^Mean pore diameter (nm).

**Figure 5:**
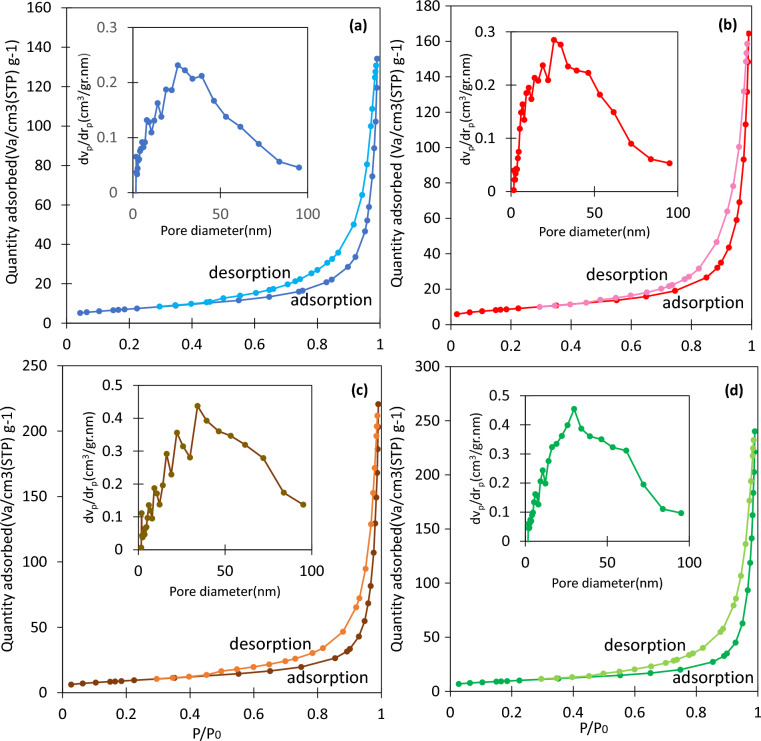
Nitrogen adsorption − desorption isotherm and BJH pore size distribution curves for the: (**a**) Co/Ni-Ferrite, (**b**) Co/Co-Ferrite, (**c**) Co/Cu-Ferrite, and (**d**) Co/Zn-Ferrite.

In Fig. 5, the nitrogen adsorption and desorption isotherms for the supported catalysts are presented. The isotherms of spinel ferrite carriers were included in the supplementary data, Figure S1. All the samples exhibit a type III adsorption isotherm. The size distribution of the pores in the inner part of each shape suggests that these samples have a mesoporous and macropores structure, most of them fall within the range of 2–50 nm. These large pores act as a bridge between the micropores, creating an additional transfer path for reactant molecules^33^.

#### FTIR analysis

**Figure 6:**
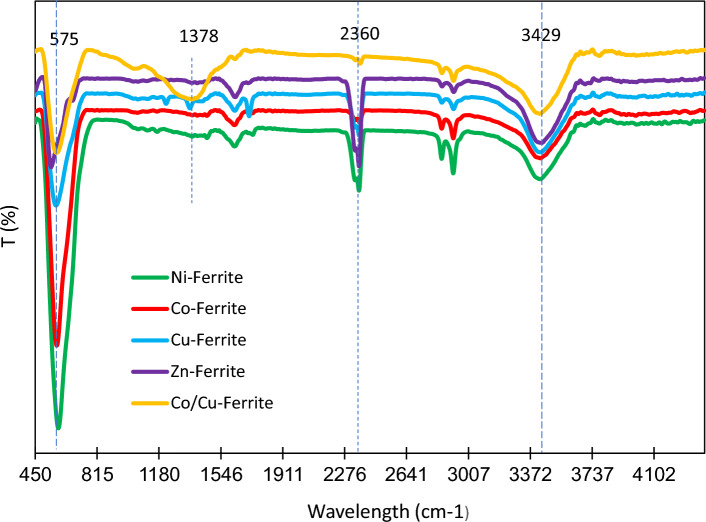
FTIR spectrums of four spinel ferrites and Co/Cu-Ferrite.

FTIR spectra were recorded for spinel ferrites and Co/Cu-Ferrite in the range of 450–4400 cm^−1^ and the results are shown in Fig. 6. The prominent absorption band at 575 cm^−1^, from the stretching vibrations of the Fe–O group, provides evidence for ferrite formation. The peak at 2360 cm^−1^ in the infrared spectrum of ferrites is typically associated with atmospheric CO_2_. The broad bands at 3429 cm^−1^ of all samples indicated the obvious presence of hydroxyl group (-OH)^34^. Compared with that of ferrite-based supports, a new absorption band appeared for the Co/Cu-Ferrite sample. Peaks between 1200 and 1400 cm^−1^ are commonly associated with metal–oxygen vibration molecules. So, a peak in this region may indicate the presence of cobalt-oxygen bonds in this sample.

### Activity test

**Figure 7:**
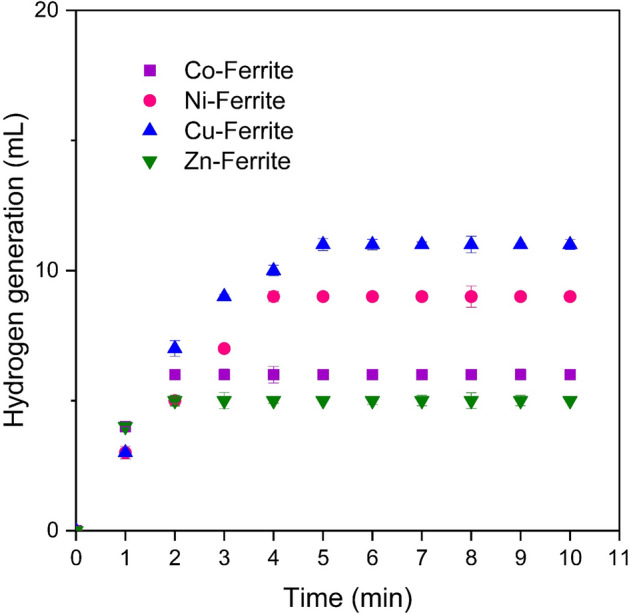
Hydrogen generation curves of the NaBH_4_ hydrolysis over different spinel ferrites.

To investigate the effectiveness of the synthesized catalysts in the process of sodium borohydride hydrolysis, the quantity of hydrogen generated via the hydrolysis of NaBH_4_ on the surface of these nanocatalysts was measured in a continuous setup utilizing the common water displacement method. The outcomes of this investigation are presented in Fig. 7. As evident from the results, the presence of bare supports resulted in a considerably low and negligible generation of hydrogen on each of the carriers. This low amount is due to the self-hydrolysis of NaBH_4_ and, it is low due to the sluggish kinetics of the self-hydrolysis process at room temperature, as much literature reported^35^. However, copper ferrite outperformed the other samples due to its smaller and more uniform particle distribution. Hence, it can be inferred that the synthesized supports do not exhibit any obvious catalytic activity in the process of sodium borohydride hydrolysis.

**Figure 8:**
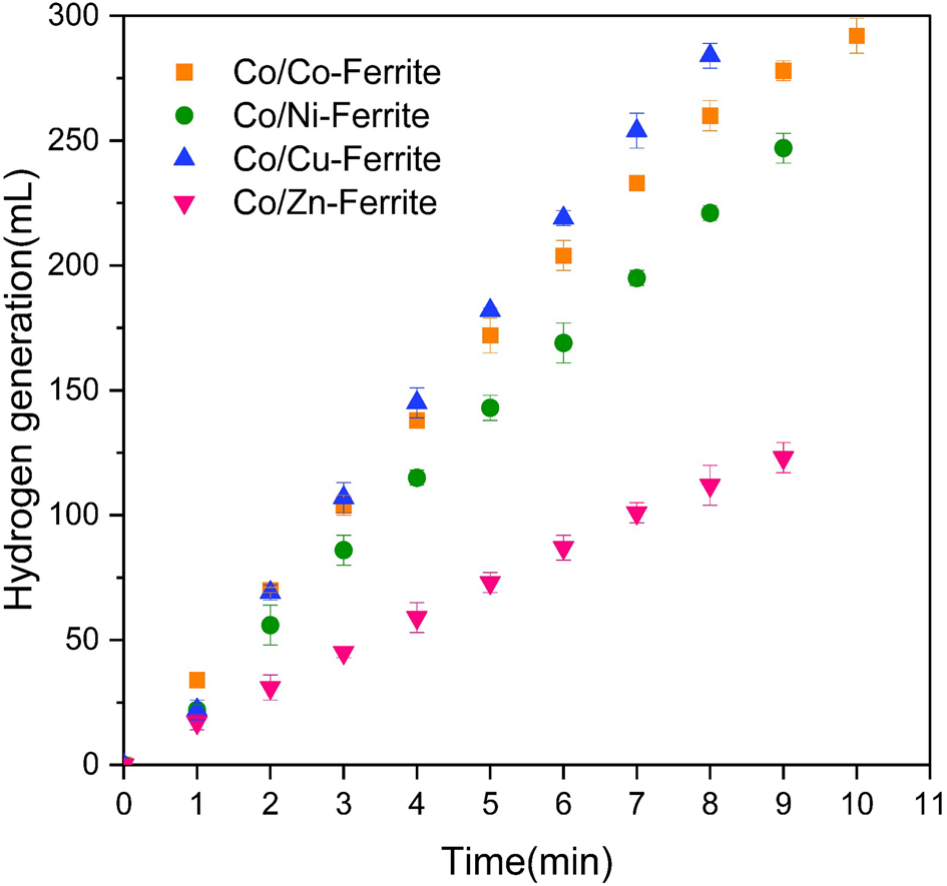
Hydrogen generation curves of the NaBH_4_ Hydrolysis over different supported spinel ferrites.

The performance of the samples after the loading of cobalt is depicted in Fig. 8. According to the results, while the bare supports exhibited little catalytic activity, the introduction of cobalt as an active phase on the substrate led to a considerable enhancement in hydrogen production, suggesting that the active component in the hydrolysis of NaBH_4_ is cobalt rather than the support. Specifically, the Co/Zn-Ferrite catalyst demonstrated the lowest amount of hydrogen generation, whereas the Co/Cu-Ferrite catalyst exhibited the highest amount. The superior catalytic performance of the Co/Cu-Ferrite sample, attaining a maximum generation rate of 2937.5 mL/min.g_cat_, can be attributed to its higher particle distribution and larger surface area compared to other catalysts. However, for other samples, their weaker pore characteristics and particle distribution resulted in lower performance. It is evident that the obtained results are consistent with the pore characteristics, especially the pore volume of the samples. In addition, research indicates that Cu-Ferrite has higher electrical conductivity and lower dielectric constant than other Ferrites^36^. A literature review has revealed that the effectiveness of a catalyst is influenced by the adequate dispersion of active components, the quantity of active sites, and the high electron density of active sites. Therefore, the hydrolysis process will perform better when the surface of the cobalt metal has a higher concentration of electrons^37^. So, Cu-ferrite with higher electron donating properties and charge carrier density can increase the electron density of the cobalt active sites, resulting in enhanced catalytic activity of the Co/Cu-Ferrite nanocatalyst during the hydrolysis reaction. Consequently, for further investigations, the cobalt catalyst supported on Cu-Ferrite was selected as the main catalyst for the hydrolysis process.

**Figure 9:**
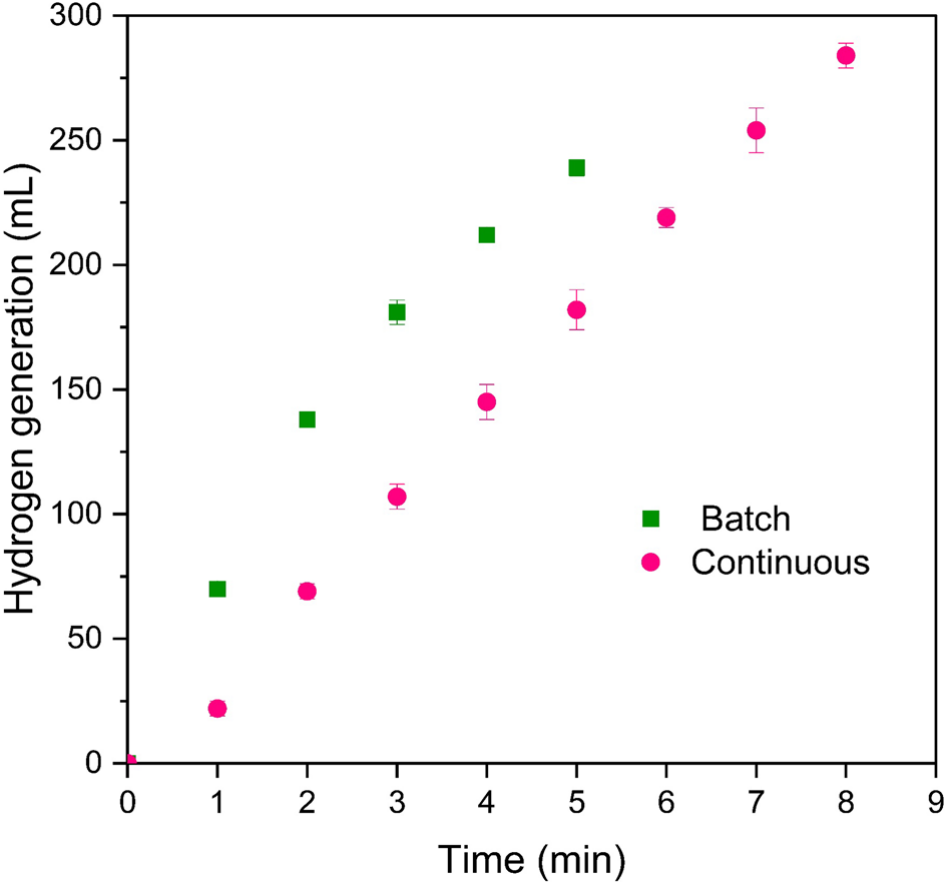
Comparison of hydrogen evolution of the NaBH_4_ hydrolysis in the batch and continuous system.

As stated earlier, the present study aimed to investigate the NaBH_4_ hydrolysis process in a continuous hydrogen generation system. Nevertheless, to evaluate the impact of the type of reactor setup on the hydrogen generation rate, the sodium borohydride hydrolysis process was conducted under the temperature of 35 °C, using 16 mg of catalyst and a 3 mL solution containing 2 wt.% NaBH_4_ and 4 wt.% NaOH, separately in both continuous and batch setups. The sole difference between these two methods was the utilization of a syringe pump with a predetermined flow rate in the continuous stirred tank reactor instead of administering the reagent solution all at once. As can be seen in Fig. 9, the batch system yielded a greater quantity of hydrogen over a shorter duration. Specifically, 138 mL of hydrogen was produced within the 2 min in the batch process, while this value was 69 mL in the continuous system. The reason why batch stops earlier than continuous can be attributed to the fact that in a batch reactor, the reactants are added all at once at the beginning of the reaction, while in a continuous flow reactor, the reactants are continuously supplied. If a reactant is depleted before completion, a batch reactor may end the reaction prematurely. At the same time, the larger amount of borohydride ion that is exposed to the catalytic active sites in the batch setup can increase the yield of production. Despite this, due to the lack of comprehensive investigations on the sodium borohydride hydrolysis process and its associated parameters in continuous setups, hydrogen generation was conducted under a continuous stirred tank reactor to further continue the research.

### Effect of different parameters on the catalyst activity

#### Effect of catalyst quantity

**Figure 10:**
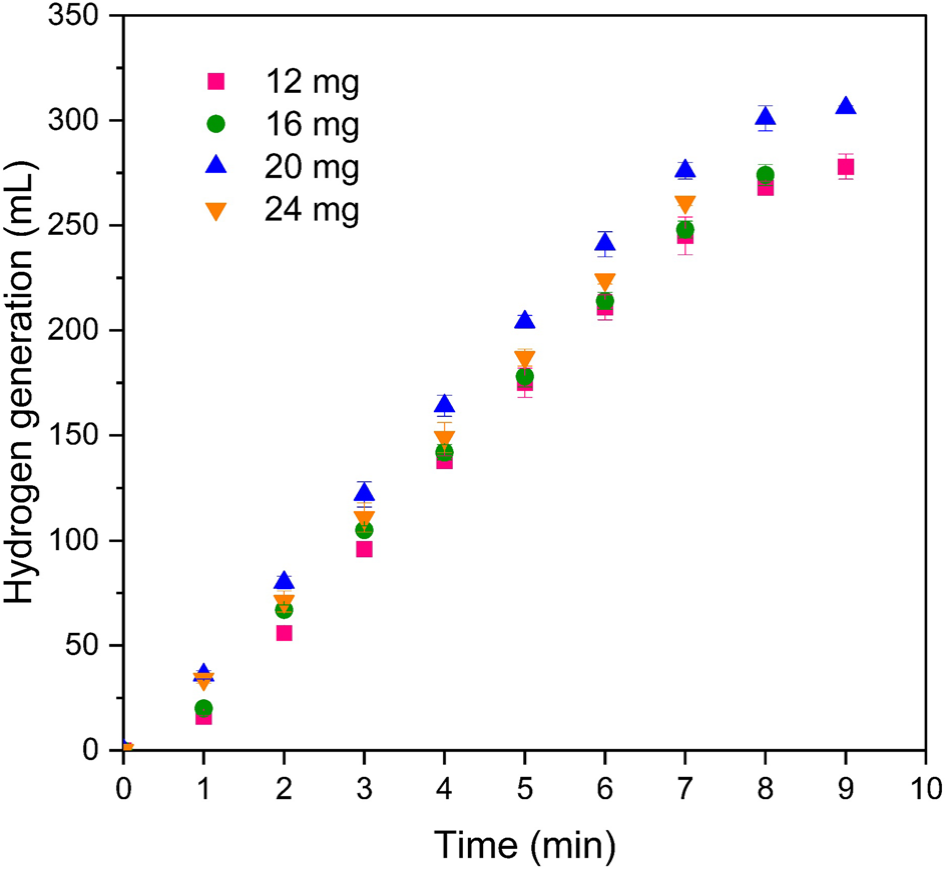
The effect of catalyst amount on the volume of hydrogen generation (2wt.%NaBH_4_, 4wt.%NaOH, 35℃, and flow rates of 30 mL/h).

The effect of catalyst dosage on the amount of hydrogen gas generated is presented in Fig. 10. As demonstrated, the quantity of produced hydrogen increased by increasing the catalyst amount, from 12 to 20 mg. However, beyond this point, with a further increase in catalyst dosage to 24 mg, the hydrogen generation rate began to decline. This phenomenon can be attributed to the fact that although increasing the quantity of catalyst leads to an increase in catalytic active sites, it simultaneously increases the solution viscosity and active site coverage, thereby diminishing the hydrogen generation volume^38^. Thus, it can be deduced that during the initial stages of the process, the rise in catalytic active sites was the primary factor in the increase of hydrogen production. However, upon exceeding a catalyst dosage of 20 mg, mass transfer hindrances lead to a reduction in hydrogen generation.

#### Effect of feed flow rate

**Figure 11:**
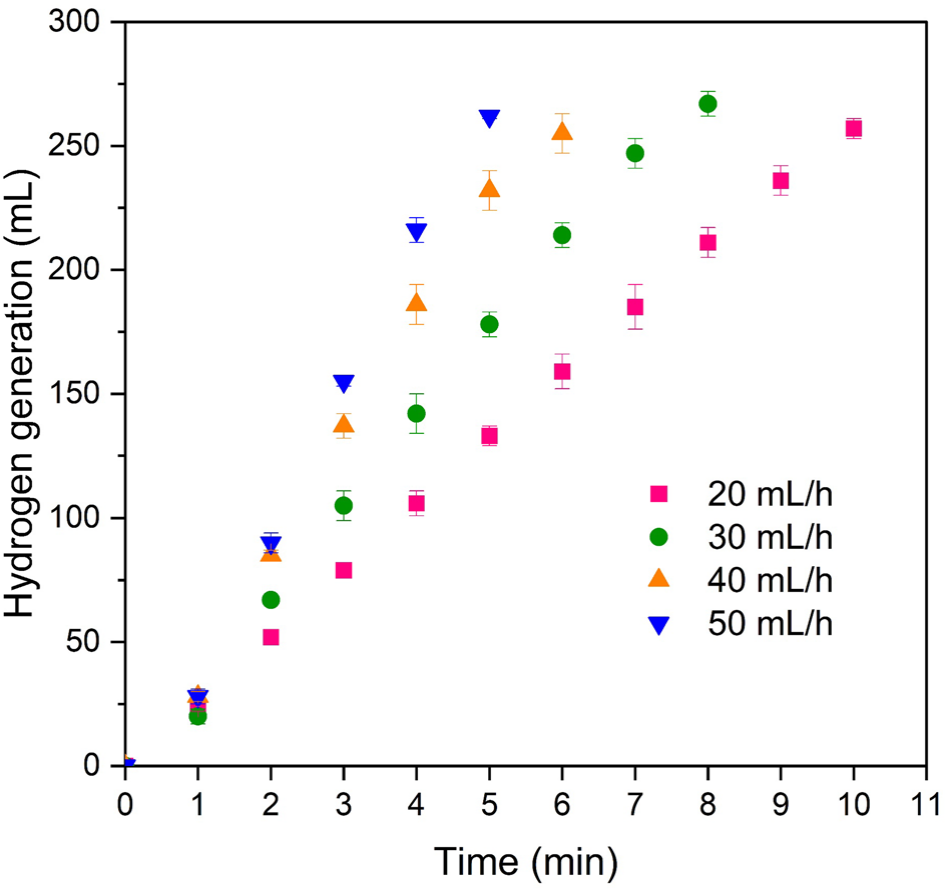
The effect of reactive solution flow on the volume of hydrogen generation (16 mg cat, 2 wt.%NaBH_4_, 4wt.%NaOH, and 35℃).

Figure 11 illustrates the impact of the flow of a reactive solution on the rate of hydrogen generation in a continuous system. The results demonstrate that hydrogen generation increases with higher flow rates due to the increased contact between sodium borohydride and the catalyst, resulting in a more substantial reaction. Additionally, it can be observed that under a consistent flow rate during solution injection, hydrogen generation also exhibits a constant rate.

In our previous study^35^, we proposed a mechanism for this process. First, BH_4_^−^ and H_2_O molecules get adsorbed on the surface of the catalyst. The BH_4_^−^ ions chemisorb to the catalyst surface, which leads to the production of CoBH_3_^−^ and CoH intermediates. These intermediates then react with H_2_O to produce BH_3_(OH)^−^ ion and H_2_. Subsequently, the hydrogen shifts from BH_3_(OH)– ion to an unoccupied cobalt atom according to BH_3_(OH)^−^  → BH_2_(OH)_2_^−^ → BH(OH)_3_^−^ → B(OH)_4_^−^ reaction. By repeating this procedure, 4H_2_ is produced at each cycle as follows:

$${\text{NaBH}}_{{4}} + {\text{4H}}_{{2}} {\text{O }} \to {\text{NaB}}\left( {{\text{OH}}} \right)_{{4}} + {\text{4H}}_{{2}}$$

#### Effect of temperature

**Figure 12:**
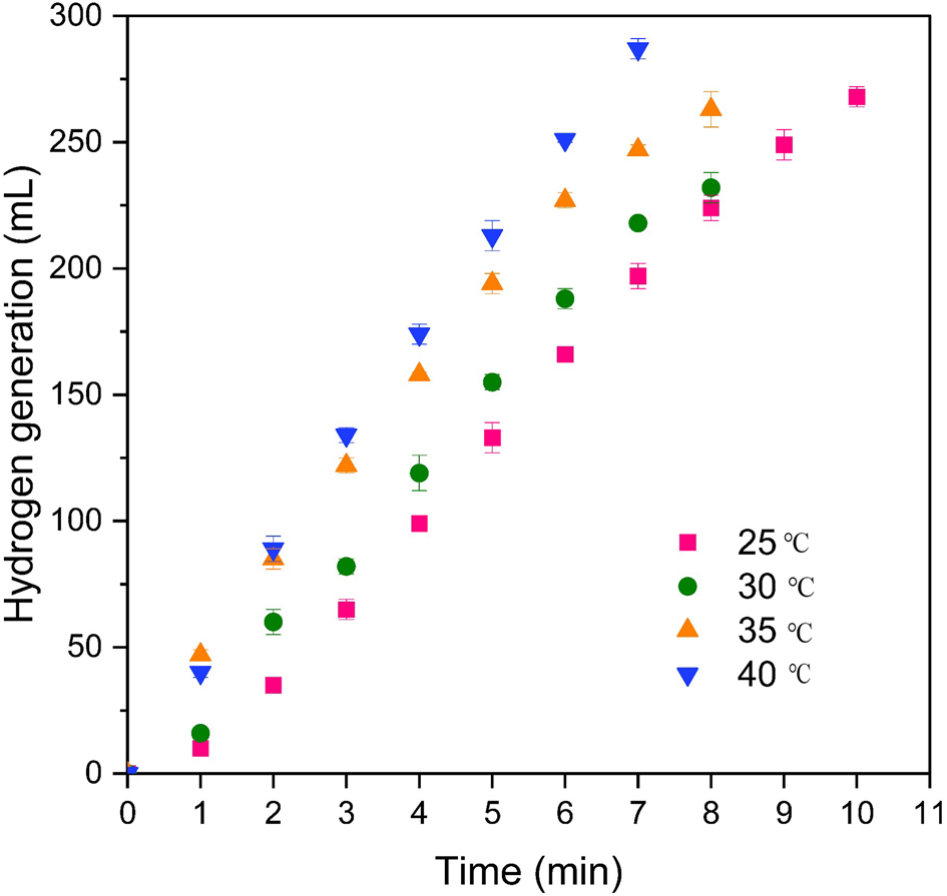
The effect of temperature on the volume of hydrogen generation (16 mg cat, 2 wt.%NaBH_4_, 4wt.%NaOH, and flow rates of 30 mL/h).

To investigate the impact of temperature on the catalytic hydrolysis of sodium borohydride, the experiments were conducted across a range of temperatures (25–40 °C), while maintaining a constant concentration of sodium borohydride, catalyst quantity, and sodium hydroxide concentration. The results, illustrated in Fig. 12 demonstrate a direct relationship between temperature and the rate of reactions in the sodium borohydride hydrolysis process, leading to an increase in the generation of hydrogen gas. A higher rate of hydrogen generation at higher temperatures is consistent with the expected behavior of molecules at higher temperatures, which tend to be more active and available.

### Kinetic study

According to numerous literature reports, the process of hydrolyzing NaBH_4_ follows zero-order kinetics^39^. This is based on the observation of a linear increase in hydrogen generation volume over time at fixed NaBH_4_ concentrations. So, the zero-order reaction calculation is used in the kinetic study. By applying the Arrhenius equation, Eqs. (1) and (2), and determining the slope of the resultant line, the activation energy can be calculated:

1 $${\text{r}} = {\text{k}} = {\text{k}}_{0} .{\text{exp}}\left( { - \frac{{E_{a} }}{R.T}} \right)$$

2 $${\text{Ln}}\left( {\text{r}} \right) = {\text{ Ln}}({\text{k}}_{0} ) - \frac{{E_{a} }}{R.T}$$

In the above equations, the symbol "r" represents the hydrogen generation rate, "k_0_" denotes the frequency factor, "E_a_" stands for the activation energy, "R" represents the ideal gas constant, and "T" signifies the reaction temperature.

**Figure 13:**
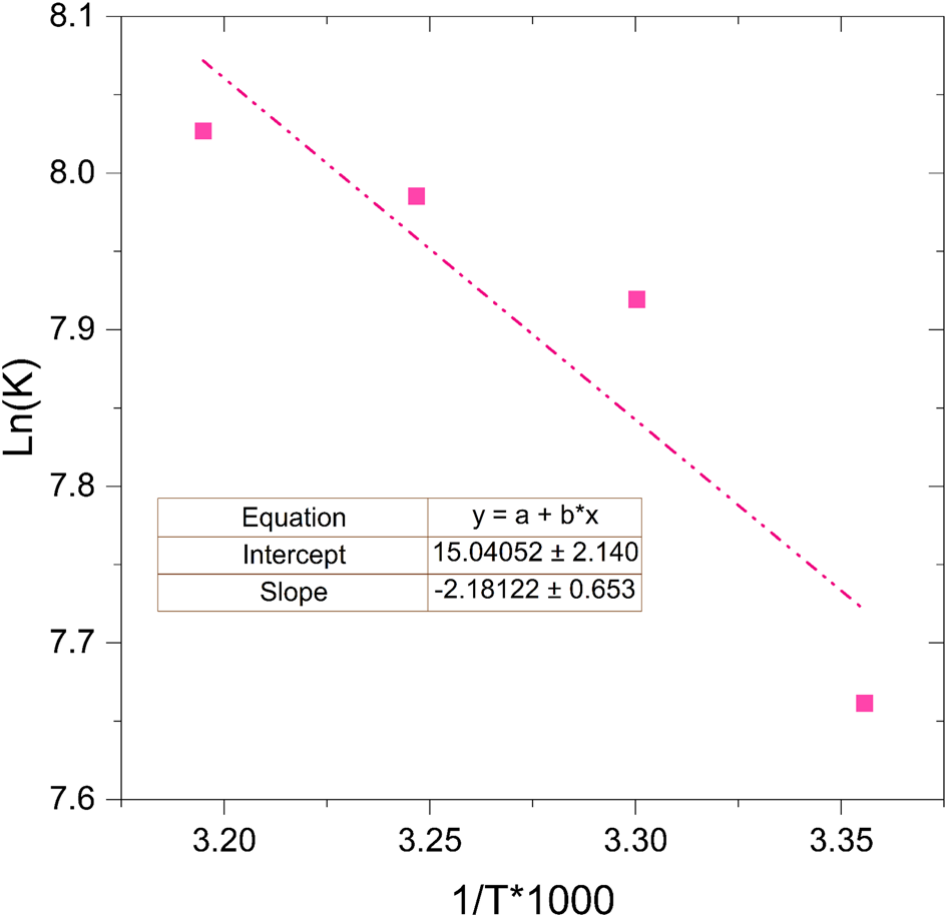
Arrhenius diagram obtained from the data in Fig. 12.

The graphical representation in Fig. 13 depicts the correlation between Lnk and 1/T, derived from the data presented in Fig. 12. By stating the slope of the resultant line, the activation energy for Co/Cu-Ferrite nanocatalyst was calculated to be 18.12 kJ/mol. However, the activation energy was calculated for other catalysts too. Figure S2 shows the correlation between Lnk and 1/T for all cobalt-based samples. Through the slope and intercept analysis of this figure, we calculated the activation energy for Co/Co-Ferrite, Co/Ni-Ferrite, and Co/Zn-Ferrite as 20.5 kJ/mol, 43.6 kJ/mol, and 55 kJ/mol respectively. Considering that the lowest activation energy was obtained on the Co/Cu-Ferrite sample, the superior catalytic performance of Co/Cu-Ferrite was confirmed again. A lower activation energy will generally result in a faster reaction rate and can facilitate the reaction more efficiently. Moreover, the obtained activation energy values are consistent with the pore characteristics of the catalysts. The relatively low activation energy on Co/Cu-Ferrite confers a positive attribute to the catalyst, as it lowers the amount of energy needed for the reaction to initiate.

### Reusability test

**Figure 14:**
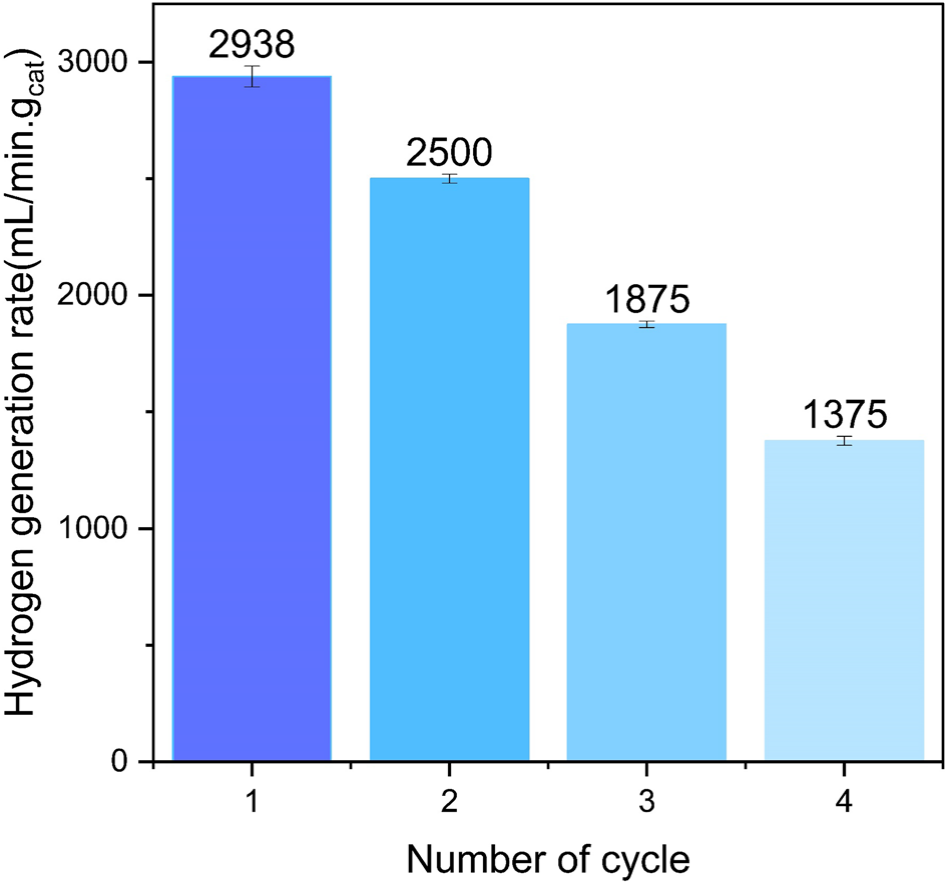
The stability of Co/Cu-Ferrite catalyst for 4 repetitive cycles.

The stability of the catalyst holds pivotal importance in the successful commercialization of hydrogen generation systems reliant on NaBH_4_ hydrolysis. To this end, the current investigation aimed to evaluate the catalytic stability of the Co/Cu-Ferrite nanocatalyst through four successive and repeated applications. The findings of Fig. 14 suggests a decrease in the catalyst's activity with each subsequent use. This reduction in activity can be attributed to the decline in catalyst mass and leaching of cobalt active phase during each wash, which has a large contribution to the catalyst activity reduction. Furthermore, the reduction in catalyst activity can be attributed to additional factors, including the accumulation of byproducts on the catalyst surface, aggregation of the metal active phase, etc.^35^.

Table 2 compares the hydrogen generation potential of different ferrite-based catalysts, evaluating their performance based on two critical parameters, namely, activation energy and hydrogen generation rate. It can be inferred that the catalyst under investigation has achieved a satisfactory outcome in comparison to the performance of the other catalysts cited, as evidenced by the results presented in Table 2.

**Table 2:** The hydrogen generation properties of different ferrite-based catalysts.

Catalyst	Temperature (℃)	Catalyst amount (mg)	NaBH_4_ concentration (wt%)	HGR (mL/min.g)	E_a_ (kJ/mol)	Ref
CuFe_2_O_4_	30	–	10	1500	41.53	^20^
CuFe_2_O_4_/RGo	25	30	45 mM	622	33.95	^40^
NiB/NiFe_2_O_4_	25	100	0.5	299.88	72.52	^18^
NiFe_2_O_4_	70	10	0.66	2181	50	^41^
Ag/CoFe_2_O_4_–CNT	25	50	3mmol	320	14.7	^19^
Co/Cu-Fe_2_O_4_	35	16	2	2937	18.12	This work

## Conclusion

The present investigation involved the synthesis of cobalt-based catalysts on nickel, cobalt, zinc, and copper ferrites for use in the sodium borohydride hydrolysis process in a continuous flow system. The catalytic activity of the synthesized samples was assessed at a temperature of 35 °C, employing 16 mg of catalyst and 3 mL of an aqueous solution containing 2 wt.% NaBH_4_ and 4 wt.% NaOH via the water displacement method. It was found that Co/Cu-Ferrite exhibited superior characteristics, resulting in a high hydrogen generation rate of 2937 mL/min.g_cat_. Higher particle distribution, electrical conductivity, and larger surface area compared to other samples are responsible for its high activity. Additionally, calculating the activation energy for all cobalt-based catalysts, the activation energy on the Co/Cu-Ferrite catalyst was determined to be as low as 18.12 kJ/mol, lower than others which verifies the superiority of the Co/Cu-Ferrite sample. This low activation energy is deemed favorable in the design and application of this catalyst in the hydrolysis reaction of sodium borohydride. However, the catalytic activity of the Co/Cu-Ferrite catalyst was observed to gradually decrease over 4 repeating applications. The decrease in catalyst mass and leaching of the cobalt active phase during each wash was identified as one of the main reasons for this phenomenon.

## Supplementary Information

Supplementary Figures.

## Data Availability

The datasets used and analyzed during the current study are available from the corresponding author upon reasonable request.
