# Supplementary material for: Enhancing hydrogen generation from sodium borohydride hydrolysis and the role of a Co/CuFe2O4 nanocatalyst in a continuous flow system
Source: Sci Rep. 2024 Apr 26;14:9659. doi: 10.1038/s41598-024-60428-5 (PMC11579333; doi:10.1038/s41598-024-60428-5)
Supplement: Supplementary file 1 — Supplementary Figures. [file 41598_2024_60428_MOESM1_ESM.docx]

**Enhancing Hydrogen Generation from Sodium Borohydride Hydrolysis and the Role of a Co/CuFe_2_O_4_ Nanocatalyst in a Continuous Flow System**

Faezeh Mirshafiee^1^, Mehran Rezaei ^[[1]](#footnote-1),1^

1. School of Chemical,Petroleum and Gas Engineering, Iran University of Science and Technology (IUST), Tehran, Iran

Figure S1: Nitrogen adsorption−desorption isotherm for the bare supports

Figure S2: Arrhenius diagram of synthesized catalysts

1. Corresponding author:

   * Mehran Rezaei, mrezaei@iust.ac.ir [↑](#footnote-ref-1)
